# Verbalizing spiritual needs in palliative care: a qualitative interview study on verbal and non-verbal communication in two Danish hospices

**DOI:** 10.1186/s12904-021-00886-0

**Published:** 2022-01-04

**Authors:** Sara Stage Voetmann, Niels Christian Hvidt, Dorte Toudal Viftrup

**Affiliations:** Research Unit of General Practice, Department of Public Health, University of Southern Denmark, Odense, Denmark

**Keywords:** Hospice, Palliative care, Spiritual care, Non-verbal communication, Sensing, Existential psychology, Philosophy of caring sciences, Theory of caring sciences, Qualitative interviews, Interpretative phenomenological analysis

## Abstract

Denmark is considered one of the World’s most secular societies, and spiritual matters are rarely verbalized in public. Patients report that their spiritual needs are not cared for sufficiently. For studying spiritual care and communication, twelve patients admitted to two Danish hospices were interviewed. Verbal and non-verbal communication between patients and healthcare professionals were identified and analysed. Methodically, the Interpretative Phenomenological Analysis was used, and the findings were discussed through the lenses of existential psychology as well as philosophy and theory of caring sciences. Three themes were identified: 1. *When death becomes present,* 2*. Direction of the initiative,* and 3. *Bodily presence and non-verbal communication.* The encounter between patient and healthcare professional is greatly influenced by sensing, decoding, and interpretation. A perceived connection between the patient and the healthcare professional is of great importance as to how the patient experiences the relationship with the healthcare professional.The patient’s perception and the patient’s bodily experience of the healthcare professional are crucial to whether the patient opens up to the healthcare professional about thoughts and needs of a spiritual nature and initiates a conversation hereabout. In this way we found three dynamically connected movements toward spiritual care: 1. From secular to spiritual aspects of care 2. From bodily, sensory to verbal aspects of spiritual care and 3. From biomedical to spiritual communication and care. Thus, the non-verbal dimension becomes a prerequisite for the verbal dimension of spiritual communication to develop and unfold. The behaviour of the healthcare professionals, characterised by the way they move physically and the way they touch the patient, was found to be just as important as verbal conversation when it comes to spiritual care. The healthcare professional can create a connection to the patient through bodily and relational presence. Furthermore, the healthcare professionals should let their sensing and impressions guide them when meeting the patient in dialog about matters of a spiritual nature. Their perception of the patient and non-verbal communication are a prerequisite for being able to meet patient’s spiritual needs with care and verbal communication.

## Background

The Danish National Board of Health focuses on patients’ spiritual needs in their recommendations for the palliative care in Denmark [1]. These recommendations are anchored in WHO’s definition of spiritual pain as one of the four health professional attention fields in their definition of total pain in palliative context alongside physical, mental, and social pain [1]. However, international research from the patients’ perspective has clarified that care for this fourth aspect of pain in palliative care has the least focus [2–4]. This might be even more the case in Denmark and the other Nordic countries which are considered some of the World’s most secularized societies, where spiritual and existential matters traditionally belong within the private spheres and rarely are addressed in institutional contexts such as healthcare institutions [5]. Hence, spiritual care in palliative care in Denmark is highly relevant to study in order to improve and develop spiritual care in palliative practice.

In nationwide guidelines, the notion of spirituality is multidimensional and includes: 1. Existential challenges, 2. Value based considerations and attitudes, and 3. Religious considerations and foundations [6]. For this study we embraced a concept of spiritual care including general psychosocial (secular), spiritual, religious, and/or existential aspects of patient support and care [1, 7–10].

Several studies have described how terminal patients, chiefly cancer patients, experience an insufficient focus on their spiritual needs in their course of treatment [5, 11–13]. Recent studies indicate how healthcare professionals (HCPs) feel obligated to try to meet the patients’ spiritual needs. However, due to respect for the autonomy of the patients and because HCPs have difficulties identifying these needs, they often refrain from talking about spiritual matters unless the patients themselves explicitly invite them to do so [14]. HCPs’ reluctance to verbally address the patients’ spiritual concerns may thus result in the patients’ need for spiritual care not being met [15]. The issue is further complicated by the fact that the patients do not necessarily expect that spiritual care can be part of their care, and therefore not tend to verbally articulate these needs [16].

It may therefore be suggested that the patients’ needs for spiritual care more frequently manifest themselves as non-verbal cues and clues in the interaction between the patient and HCP, and how HCPs notice and act upon these cues and clues may determine whether conversations of a spiritual nature arise or not. Even though there have been efforts made to improve palliative care and providing spiritual care, conversations on spirituals matters remain a challenge to many HCPs [17, 18]. A lack of knowledge on how to initiate a conversation as well as uncertainty about doing so are significant barriers [17]. Spiritual care can be considered a relational, ongoing process which holds the potential for mutual development and change for both the patient and HCP. This while, in interaction, examining, talking about, and addressing the patients’ spiritual needs and matters [18]. It is stated, that neglecting the patients’ needs, with the risk of subsequent spiritual suffering, can be an unforeseen negative consequence of the HCPs not being in touch with their own spirituality. In addition to benefiting the patients, a desirable self-awareness- and reflection seems to have a protective effect on the HCPs both on an individual level and so that they do not become reluctant in meeting the patients’ existential and spiritual needs. This is one of the reasons why systematic education in spiritual care is recommended in both the United States and in Europe [17, 18]. The purpose of this study was hence to investigate how spiritual matters are communicated both verbally and non-verbally in the interaction between patient and HCP in two Danish hospices.

## Method

For studying this purpose, we applied the qualitative method Interpretative Phenomenological Analysis (IPA) [19]. IPA originates from health psychology but is used increasingly within health sciences. IPA is developed to examine how people create meaning based on their experiences and IPA is phenomenological in the sense that it can be used to describe experiences with the participants’ own words. Therefore, IPA seemed to be an appropriate approach for the purpose of the study. Based on an interview guide, we conducted 12 semi-structured interviews at Hospice Sydfyn on the South of the island Funen and Arresødal Hospice on the North of the island Sjaelland. Purposeful sampling was performed and participants were recruited in collaboration with the HCPs at the hospices [19]. Based on a personal knowledge and a clinical assessment of possible participants, the HCP on duty asked if the patient might be interested in meeting the first and the third authors, respectively. If the patient agreed to this, they were introduced to the purpose of the study and their possible participation in a qualitative research interview. Thereafter, the patient could either consent to participation or refuse to participate. All interviews were recorded and transcribed in a pseudomized manner, after which the audio files were deleted. The first author conducted three interviews with patients, while the last author conducted nine. The interviews lasted, depending on the patient’s condition, between 19 min and 56 min (average 36 min). The interviews took place in the patients’ rooms at the hospices. Eight women and four men participated, and the average age was 61 years. The patients were all diagnosed with different kinds of terminal cancer.

In addition to being a method of conducting research, IPA is also an analytical strategy for analyzing interview data [19]. The analysis was performed step-by-step. First, the interviews were transcribed by the first and last authors, respectively. Thereafter, data were provided with notes on both the content and structure of the interviews and an incipient interpretation of data took place. In the next step, data, which now consisted of both interview data and interpretive notes, were condensed into themes, which eventually became overarching themes and sub-themes. This process was conducted for all interviews separately. Finally, the interpretive, step-by-step analysis consisted of studying patterns across the 12 interviews and developing final themes for the entire dataset.

### Theory

For interpretation of the findings, we used theory of theologian and philosopher K.E. Løgstrup [20], professor of nursing Kari Martinsen [21–23], and professor of psychology Bo Jacobsen [24]. Løgstrup describes how in all human encounters there is an unspoken demand to take care of the person who meets us. The demand stems from a basic interpersonal trust and in the encounter with another human being the most responsible way to respond to this trust is based on a belief in what is best for the other [8, 20]. Kari Martinsen, like Løgstrup, also focuses on relational, interpersonal relationships, and the sensing that the human encounter brings about. She describes this as *interpretation*. Interpretation consists in sensing and interpreting the other human being in the relation through what the other expresses both verbally and bodily. Sensing makes us receptive to impressions from other people [21–23]. Bo Jacobsen’s existential psychology is based on existential philosophy and focuses on how humans think and relate to life and the basic conditions and questions in life (e.g. death, freedom, isolation, and meaning) in relation to their daily lives [24].

### Findings

Through the step-by-step, interpretive analysis, three themes emerged: 1. *When death becomes present,* 2*. Direction of the initiative,* and 3*. Bodily presence and non-verbal communication*. Below we present these themes, and subsequently these are discussed in relation to the three theoretical perspectives.

#### When death becomes present

The patients all reflect on and want to talk about death, including their own impending death. Death becomes, so to speak, present for the patients. For example, a patient expressed how a gradual habituation takes place, where death becomes integrated as part of everyday life:

*“And it [death] had a lot of focus for me in the beginning. I may have gotten use to it by now, I think. Although it doesn’t make it better, though. I think I have gotten use to it. A little bit of everyday life has come into it.”*

This quote illustrates, on one hand, how death becomes a part of everyday life. Conversely, the quote supports that, although death is present for the patient, she also maintain the importance of everyday life containing more than illness and being a patient. Several of the patients emphasized everyday life and being self-reliant as important. One patient put it this way:

*"I'm so tired of always being the weak one. And, I have been [weak] for so long. It happens when you are hospitalized often. Then you become nothing but a patient; anonymous. Education and personal interests, everything just disappears […] I get up every day, take a bath and get dressed… I need it to maintain myself […] my children, my family are important to me and I’m also very involved in my hobby. I still do all these things even though I'm here.”*

This quote shows how the patient strive to integrate the presence of death, without it becoming overriding and dominant while maintaining herself and everyday life. It seems that having a daily life despite the presence of death is an essential need among the patients. Furthermore, it also appears as a significant spiritual need for the patients, that both themselves and their surroundings perceive them as more than just ill patients.

Just as the presence of death influences the patients’ everyday lives, it is seen that their perception of time, and the way they seem to be aware of their being in time, are affected by the presence of death. As an example, one patient stated:

*“I don’t want to know,‘you have six month or you have this or that’. So, every time I meet a new doctor, I say, ‘no prognosis’."*

This quote illustrates how this patient reflects on the impact time has on her existence and on the conversations she has with the doctors. The impact time has on existence seems to facilitate a consciousness on the fact that the patients are forced to prioritize their time. Furthermore, time can also be perceived as a facilitator in relation to completing essential life tasks and making important decisions. One patient put it this way:

*"I think it's good to talk about it, because my son was really angry that we had to… or that we have talked about how I felt if I had a cardiac arrest. […] But, as I said, ‘it can’t help that we wait a month’."*

Another patient stated:

*"There are many things I would like to finish. […] It is a recognition of the fact that I am, where I am, right? And, I’ll take it as it comes. There’s nothing else to do about it.”*

These statements from patients illustrates how time, and especially the patients’ perception of their ‘limited’ time, affects their everyday lives and because their time is limited, time becomes a significant aspect of how death becomes present to them. The presence of death makes it difficult for the patients to maintain themselves in their daily lives. Patients experience that death becomes present in everyday life, but with a new understanding of time that is particularly characterized by time being limited. It is likely that the patients’ spiritual needs also include a desire to talk to the HCPs about how the presence of death influences and affects their perception of time, daily lives, and maintaining themselves.

#### Direction of the initiative

Another finding is the question of who should initiate conversations about spiritual matters and needs, for example, when death becomes present. We described this theme as ‘direction of the initiative’. The patients all expressed how they would like to talk about their thoughts and reflections of spiritual nature. However, we find a dilemma in the patients’ descriptions between, on the one hand, their wish to talk about spiritual issues and, on the other hand, uncertainty about who should initiate spiritual conversation. One patient said:

*“Interviewer: What do you think, would it be okay with you if they [the HCPs] for example asked or told you what they believe in?*

*Patient: The staff? Yes. They are welcome to do so. However, I haven’t talked about these issues with any of them.*

*Interviewer: No. No? How can that be, do you think?*

*Patient: Well. We haven’t had any needs [for discussing these issues, because they as a family already have talked about it]. I don’t know why they haven’t asked. They don’t know that we have talked about it at home."*

This quote illustrates that despite the patient’s desire to talk about spiritual matters and having done so at home, she does not find that the HCPs verbally invite to these conversations, and a dilemma arises as to who should initiate spiritual conversation. We find that some patients find it easy to initiative conversations about spiritual matters, while others wait for the HCPs to initiate a conversation. Based on these patients’ statements, it seems, that both patients and the HCPs find it difficult to transcend the dilemma of who should initiate the conversation – what direction the initiative should take, so to speak. A patient stated:

*"I feel like, if I’d like to talk, I’d almost rather ask them. Of course, they are welcome to ask me, if I would like to talk about it, then I could say yes or no, myself. But usually, I am very open to talk about it."*

This duality, in the patient’s statements, illustrates the dilemma of the direction of the initiative; some of the patients seem to prefer to take the initiative for conversation themselves, whereas other patients want the HCPs to take the initiative. If the patients themselves do not take the initiative and initiate conversation with the HCPs, our findings show that it is pivotal for the HCPs to listen to the patient’s non-verbal invitation, cues and clues, and then verbally seize the initiative and interrogate, if dialogues about spiritual matters are ever to emerge. For example, a patient said:

*“So, I really like to talk, but I'm not always that good at starting conversations myself. It's like someone has to get me started.”*

Our study suggests that this exactly point is of great importance because the patients’ need for conversations about spiritual matters often comes to their awareness, when the HCPs takes the initiative to conversation.

#### Bodily presence and non-verbal communication

In the encounter with the HCPs, the patients described how they relate to some bodily and sensory dimensions. The patients use a sort of decoding in which they try to sense and decipher whether they will be accommodated, if they initiate a conversation about spiritual matters. One patient explained it this way:

*“Interviewer: You said that about chemistry before? That there is someone you talk to better than others?*

*Patient: Yes. Yes.*

*Interviewer: What do you think makes the difference?*

*Patient: There are just some, where there is such a little more… not ten steps’ distance, but where I feel like, it is not her. Though… I have only experienced it with one here [at the hospice], where I thought our chemistry was not the best. She is nice when we talk about how I feel, and she has helped me to take a bath and she is really nice. But those slightly deeper issues – it’s not with her I would like to discuss them.”*

This quote illustrates that, in the encounter between patient and HCP, the patient is balancing which thoughts and matters the patient choses to share with the HCP. There is, so to speak, a modulation in the situation, where the patient’s spiritual reflections and thoughts appear to be more private than the caring situation itself. The patient’s decoding and sensing of the HCP becomes crucial to the extent to which the patient feel accommodated, and in confidence choses to share issues of spiritual nature with the HCP. When patients were asked to elaborate on why they felt some HCPs were better at encountering them existentially than others, they described how a bodily and non-verbal dimension particularly affected their experience of the HCPs’ availability and presence. One patient stated:

*"Well, it's just their way of being. Their way of entering the door and sit down, maybe, next to me on the bed, and putting their hand on my hand. Or, take me by the shoulder. And the way they can then ask, ‘how are you?’. And then I can hear, it’s not just, ‘do those pills work?’. Then, that’s the whole thing they’re thinking about.”*

This quote illustrates how the patient decodes the bodily actions of the HCP. Through the bodily element, the patient senses that the HCP cares for her as a whole human being. This can be described as bodily and non-verbal language, which the patient decodes and interprets. The patient feels accommodated and taken care off, because the HCP enters the door as she does, sits down, touches the patient’s hand or shoulder, and asks for her well-being in the way, she does. What the patient senses when encountering the HCP is of great importance as to how the patient experiences the relationship with the HCP. The patients describe a relational presence and a kind of connectedness in the situations where the HCPs, for example, probes not only their somatic well-being but also some more existential issues. This perceived connectedness may also illuminate the dilemma surrounding the direction of the initiative, because the way the patient senses and decodes the HCP has an impact on whether the patient opens up about thoughts and needs of a spiritual nature and thereby initiates a conversation. From the patients’ perspective, it seems that the non-verbal language with the bodily sense of presence takes precedence over the verbal dimension of communication. Also, we found that the patients gave the impression of sending some sort of signals, cues, or clues that called for the HCPs’ perception, decoding, and response. When HCPs responded to these signals, the patients experienced themselves as seen and understood, without necessarily being verbally explicit about their wishes and needs. One patient expressed this as follows:

*"And I’m not sure, that I thought the need [to talk] was there, but then they might ask me and we’ll talk anyway.”*

The patient expresses that the HCPs senses some non-verbalized needs, and when they ask deeper into what they sense, conversations of a spiritual nature can arise. This can be interpreted as an expression of the fact, that when the HCPs dares to ask to their sensation of the patient, then the patient is left with a feeling of being seen and understood. By extension, it seems that the patients emphasize the human qualities of the HCPs when describing the experience of spiritual care. One patient said:

*"They are so nice… and I also think they like me. I like to talk… that's how I’ve always been […] they are good to me and very nice.”*

This quote supports that the human qualities of the HCPs and the patient’s experienced relation with the HCPs seems to be of great importance, and that the sensing and the decoding of non-verbal communication that takes place between the patient and the HCP, is significant to the patient’s experience of spiritual care.

## Discussions

A finding in our analysis is the relational element that includes relating to the presence of death (theme 1*. When death becomes present*). Existential psychologist Bo Jacobsen describes the encounter with death and the process of finishing life with a relational element. He describes a presence or a way of being, where the dying person, in the process of finishing life, can be in companionship with, for example, close relatives or relevant HCPs [24]. The patients in our study expressed the value of the HCPs sitting with them and being present both physically and relationally. We believe that the patients’ descriptions of bodily presence and their experience of a connectedness with the HCPs can be interpreted as representations of this presence or way of being. We found no clear answer as to how the patients and HCPs relationally can transcend the dilemma surrounding the direction of the initiative (theme 2*. Direction of the initiative*), in terms of initiating conversations about spiritual matters. However, we found that the patients’ interpretation of the relation and sensing (theme 3. *Bodily presence and non-verbal communication*) is of great importance as to how the patients experiences the HCPs’ accessibility, and that a kind of connectedness is important to whether the patients opens up for conversations of a spiritual nature. Thus, we see a movement or dynamic flow from secular to spiritual aspects of care within the caring situation and in the encounter between patient and HCP. Based on this study, we will encourage HCPs to be aware of the fact that the non-verbal language seems to serve as a communication path for the patient’s wish and need for both conversations of a spiritual nature and their desire for the HCP to be *with* them in the encounter with death. Our study suggests that this being *with* the patient is essential if the HCPs should let themselves flow with the patient from a secular to a more spiritual kind of care. Dynamically connected to the flow from secular to spiritual aspects of care, we found a flow from bodily and sensory to verbal aspects of spiritual care, where the patient’s sensing, decoding, and interpretation of the HCP becomes essential to whether conversations of a spiritual kind, eventually, will arise. Eventually we see a flow fra biomedical aspects of care to again spiritual aspects of care, where asking to the patient’s wellbeeing in a certain way is not only asking to her or his somatic wellbeing and e.g. pain but also to the patient as a whole including existential and spiritual issues. Our study suggests that this approximation or flowing movement is reciprocal and therefore likewise occurs within the HCP when encountering the patient. More specifically, we would argue that HCPs should be aware of how important the non-verbal and sensory element seems to be when it comes to conversations about spiritual matters and spiritual care, and that they as HCPs dare to take the initiative and invite the patient to talk. By not responding to the patient’s cues and clues towards such conversation, HCPs potentially limit the fulfilment of their patients’ essential spiritual and existential needs.

In his philosophy of care, Løgstrup describes how in all human encounters there is an unspoken claim to take care of the person who encounter us [20]. According to Løgstrup, this claim can be expressed verbally or through actions with a potential risk that the patient’s claim will not be met, perhaps misinterpreted, or overheard by the HCP. According to Løgstrup, it will leave the patient exposed, when the claim is not answered, and the patient will then blame the HCP. In our study, none of the patients expressed disappointment or blamed the HCPs for not inviting or initiating conversations about spiritual matters. We found the exact opposite. The patients expressed how they sometimes only became aware of their need for conversations of a spiritual nature, when the HCPs asked into what they had sensed about the patient. This underlines the complexity of the dilemma surrounding the direction of the initiative and underlines the importance of daring to express and ask as to the impressions from the patient. This creates the connection to the patient that seems to be a prerequisite for a conversation to develop.

Kari Martinsen describes the body as sentient and talking in an intersubjective relationship with others. Interpretation means being present with a sentient openness to what it is like to be human, and the sensing makes us receptive to impressions [21]. We found that the bodily sensation of presence and sensing seems to precede the verbal dimension of spiritual care and communication. Again, we glimpse a flow from bodily and sensory to verbal aspects of spiritual care. This may mean that the behavior of the HCPs, the way they move and touch the patients, is of equal importance as verbal communication when it comes to spiritual care. This, for example, appears in the intersubjective relationship in the caring situation, where the patient tries to decode and decipher whether the HCP will accommodate the patient, if the patient wants to initiate a conversation, and whether the HCP has ears and gaze for the patient’s non-verbal invitation. This understanding suggests that HCPs should surrender to a sentient openness, and let their sensing and impressions guide them when meeting the patient and hereby flow *with* the patient in a movement from secular to spiritual aspects of care. Through a bodily experiences and relational presence, the HCP can connect with the patient, and thus create conditions for conversations of a spiritual nature.

Bodily presence and the perceived connectedness are important due to the patient’s interpretation of the situation and sense of the HCP and is essential to whether the patient open up and chose to share thoughts and reflections of spiritual matters. On the other hand, is seems to be important that the HCPs themselves is aware of the sensory element, when meeting the patient, and dares to ask into their impressions of the patient and hereby dares to take the initiative and initiate a conversation about spiritual matters. Because a great deal of the communication between patient and the HCP is non-verbal, there is a risk that the patient will not have her/his needs expressed and the HCP will not recognize and respond to the patient’s non-verbal invitation. It is therefore essential to be aware of the fact, that this dilemma exists in order to be able to meet the patient’s spiritual needs, for example, by daring to trust one’s impressions of signals, cues, and clues, and initiate conversations.

To sum up, we found that there is an ontological flow from 1. Sensory to verbal aspects of spiritual care as well as from 2. Secular to more spiritual aspects of spiritual care and finally from biomedical to spiritual care. Spiritual care is an art as it is a human science and must be viewed in its entirety as an aspect of palliative care that is deeply integrated into the personal and professional levels.

### Limitations

With the choice of the qualitative method IPA, it was not the intention of this study to produce knowledge that could be reproduced in a different context. The aim of the study neither is to state empirical generalizations, but to contribute to describing and illuminating a specific focus of the research field that relates to spiritual communication and care within palliative care. However, it seems plausible to assume, that the findings of this study could be transferred to other hospice patients in similar contexts. Through the analysis, it became clear that the dilemma surrounding the direction of the initiative seems to be significant when it comes to conversations of a spiritual nature. The patients are the fundation of this study and only patients were interviewed. If we, in this study, had also interviewed the HCPs or maybe used a different method, this specific issue could potentially have been elucidated in a more detailed way. It can be argued that, for example, a communication theoretical approach could have contributed to a more practical perspective when it comes to examining both verbal and non-verbal spiritual communication and to shed light on the dilemma surrounding the direction of the initiative. On the contrary, we will argue for the chosen method and the suitability of the theory, as the encounter between hospice patients and HCPs is to a great extent influenced by sensing, decoding, and interpretation. In addition, the sensory and bodily element seem to be at least as important as the verbal communication when it comes to spiritual care.

## Conclusion

Spiritual care is highly relevant to study especially in a secular setting like Denmark, where spiritual matters rarely are addressed in public and institutional contexts. Research has described how patients experience an insufficient focus on their spiritual needs in their course of treatment [5, 11–13], even HCPs feel obligated to try to meet the patients’ spiritual needs [14]. With the patients as the underlyingbasis both verbal and non-verbal communication between patients and HCPs were identified and analysed. Through the step-by-step, interpretive analysis, three themes emerged: 1*. When death becomes present,* 2*. Direction of the initiative,* and 3*. Bodily presence and non-verbal communication*. Considered through the lenses of existential psychology as well as philosophy and theory of caring sciences our study found and points out that the relation between patient and HCP is greatly influenced by sensing, decoding, and interpretation. The patient’s perception and bodily experience of the HCP seems to be crucial to whether they move forward toward the HCP and initiate a conversation about spiritual matters. This study hypothesize that the same seems to apply to the HCPs. Overarching we found an ontological flow from 1. Sensory to verbal aspects of spiritual care as well as from 2. Secular to more spiritual aspects of spiritual care and 3. From biomedical to spiritual care and that spiritual care must be viewed in its entirety as an aspect of palliative care. Thus, the non-verbal dimension becomes a prerequisite for the verbal dimension of spiritual communication to develop and unfold. The same holds for the sense of connection: The perceived connectedness between the patient and the HCP is of great importance as to how the patient experiences the relationship with the HCP. The way patients sense and decode the HCPs has impact on whether, and how patients open up to the HCPs about thoughts and needs of a spiritual nature and thereby initiates a conversation. Therefore, the dilemma surrounding the direction of the initiative and how the spiritual conversation can be initiated is essential. The HCP’s perception and non-verbal communication are a prerequisite for being able to meet patient’s spiritual needs with care and verbal communication. Our study argues that the behavior of the HCPs, the way they move and touch the patient, is just as important and inviting as the verbal conversation about spiritual matters when it comes to spiritual care. HCP can create a connection to the patient through bodily and relational presence, and the HCP should let their sensing and impressions guide them, when meeting with the patient about conversations and matters of a spiritual nature.

## Data Availability

The datasets generated and analysed during the current study are not publicly available due to small samples and data protection but are available from the corresponding author on reasonable request.

## Abbreviations

HCP(s): Healthcare professional(s)
IPA: Interpretative Phenomenological Analysis
